# Filtering the NMR Spectra of Mixtures by Coordination to Paramagnetic Cu^2+^

**DOI:** 10.1021/acs.analchem.2c01983

**Published:** 2022-07-27

**Authors:** Juan Correa, Ana Garcia-Barandela, Llorenç Socias-Pinto, Eduardo Fernandez-Megia

**Affiliations:** Centro Singular de Investigación en Química Biolóxica e Materiais Moleculares (CIQUS), Departamento de Química Orgánica, Universidade de Santiago de Compostela, Jenaro de la Fuente s/n, 15782 Santiago de Compostela, Spain

## Abstract

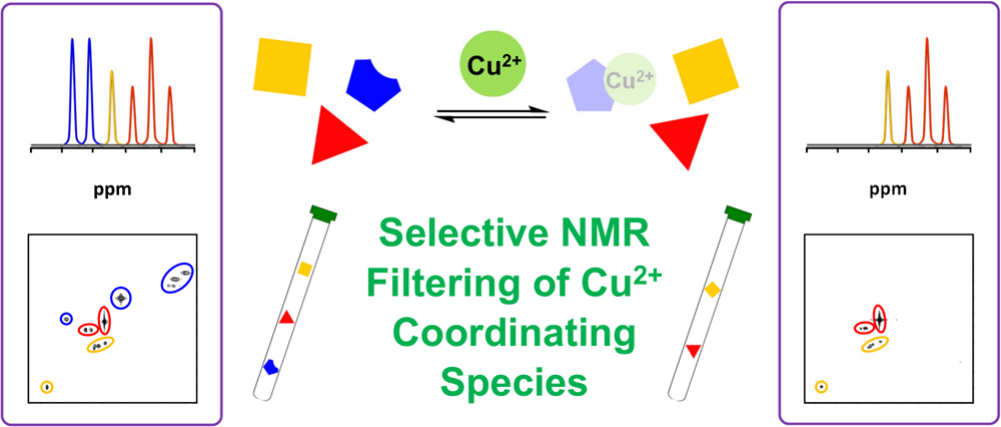

The paramagnetic spin relaxation (PSR) filter allows the selective NMR signal suppression of components in mixtures according to their complexation ability to a paramagnetic ion. It relies on the faster relaxation of nuclei in paramagnetic environments and thus is complementary to classical diffusion and relaxation filters. So far, the PSR filter has established Gd^3+^ as the sole PSR agent, restricting the paramagnetic filtering repertoire. Herein, we present Cu^2+^ as a robust PSR agent with characteristic filtering properties. While Gd^3+^ depends on unspecific ion-pair interactions with anionic components, Cu^2+^ stands out for filtering species via ordered coordination complexes. An evaluation of the paramagnetic effect of Cu^2+^ over more than 50 small molecules and polymers has unveiled different sensitivities to Cu^2+^ (especially high for pyridines, diamines, polyamines, and amino alcohols) and precise filtering conditions for mixtures (^1^H, COSY, and HMQC) that were challenged with a test bed of commercial drugs. The advantage of integrating Cu^2+^ and Gd^3+^ for the stepwise PSR filtering of complex mixtures is also shown.

As nature seldom provides pure compounds, chemists have devoted great efforts to the separation of complex mixtures. Fortunately, the NMR analysis of mixtures sidesteps the necessity of physical separations under certain conditions. NMR filters take advantage of differences in the diffusion coefficients and relaxation times of the components for the selective signal suppression of small molecules and macromolecules, respectively.^1,2^ With the aim of widening the NMR filtering portfolio beyond molecular weight limits, our group has described the paramagnetic spin relaxation (PSR) filter,^3,4^ which relies on the faster relaxation of nuclei in paramagnetic environments.^5,6^ The addition of minute concentrations of Gd^3+^ (paramagnetic) to mixtures allows the selective suppression of particular components from the 1D and 2D spectra, according to their Gd^3+^ complexation ability (Figure 1). Since Gd^3+^ has the largest spin moment (*S* = 7/2) and a high electronic correlation time (τ_s_ ca. 10^–8^ s), the Solomon–Bloembergen–Morgan equations predict a selective decrease of transverse relaxation times (*T*_2_) for species in fast chemical exchange with Gd^3+^.^7−9^ The inverse proportionality between *T*_2_ and the spectral line width^10^ leads to their selective signal suppression, without affecting the resolution and chemical shift of other components in the mixture. Since complexation to Gd^3+^ is mainly electrostatic (ion-pair), the PSR filter affects more anionic species than neutral and cationic species. Successful applications of the PSR filter include the sequential NMR filtering of mixtures of interest in the pharmaceutical and food industries^4,11^ and the fast screening of DNA ligands.^12^ Interestingly, the PSR filter benefits from a rather low sensitivity to size and molecular weight that makes it compatible with classical relaxation and diffusion filters. The advantage of this is taken for the filtering of lower PSR-sensitive species, which leads to line-broadening rather than a full signal embedment in the baseline, by implementing a complementary short *T*_2_-filter, such as the Carr–Purcell–Meiboom–Gill (CPMG).^13,14^

**Figure 1:**
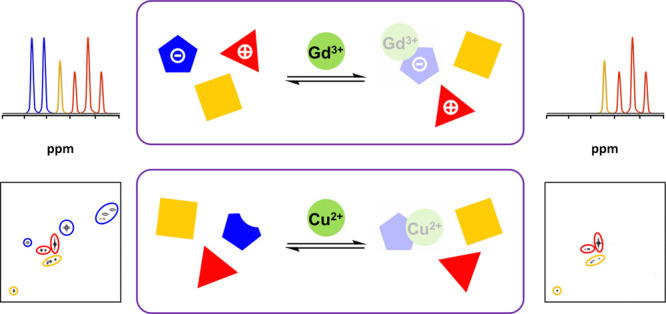
PSR filters based on Gd^3+^ (ion-pair) and Cu^2+^ (coordination) complexes.

While the PSR filter based on ion-pair Gd^3+^-complexes is highly efficient in simplifying the NMR analysis of complex mixtures, the development of PSR filters with other paramagnetic ions displaying alternative complexation modes would greatly extend the utility of this technology. Herein, we describe our efforts toward a PSR filter based on the more coordinating Cu^2+^ (*S* = 1/2, τ_s_ ca. 10^–9^ s), a paramagnetic ion aimed at selectively filtering the NMR signals of species via coordination complexes (Figure 1).

The feasibility of a Cu^2+^ PSR filter was confirmed by the selective suppression of glucosamine in the presence of glucose (Figure 2C), a suppression unfeasible to reproduce in the presence of Gd^3+^ (Figure S4) because of the similar sensitivity of both components to this ion.^4^ Conversely, the coordinating 1,2-amino alcohol moiety of glucosamine ensures a selective filtering in the presence of Cu^2+^.

**Figure 2:**
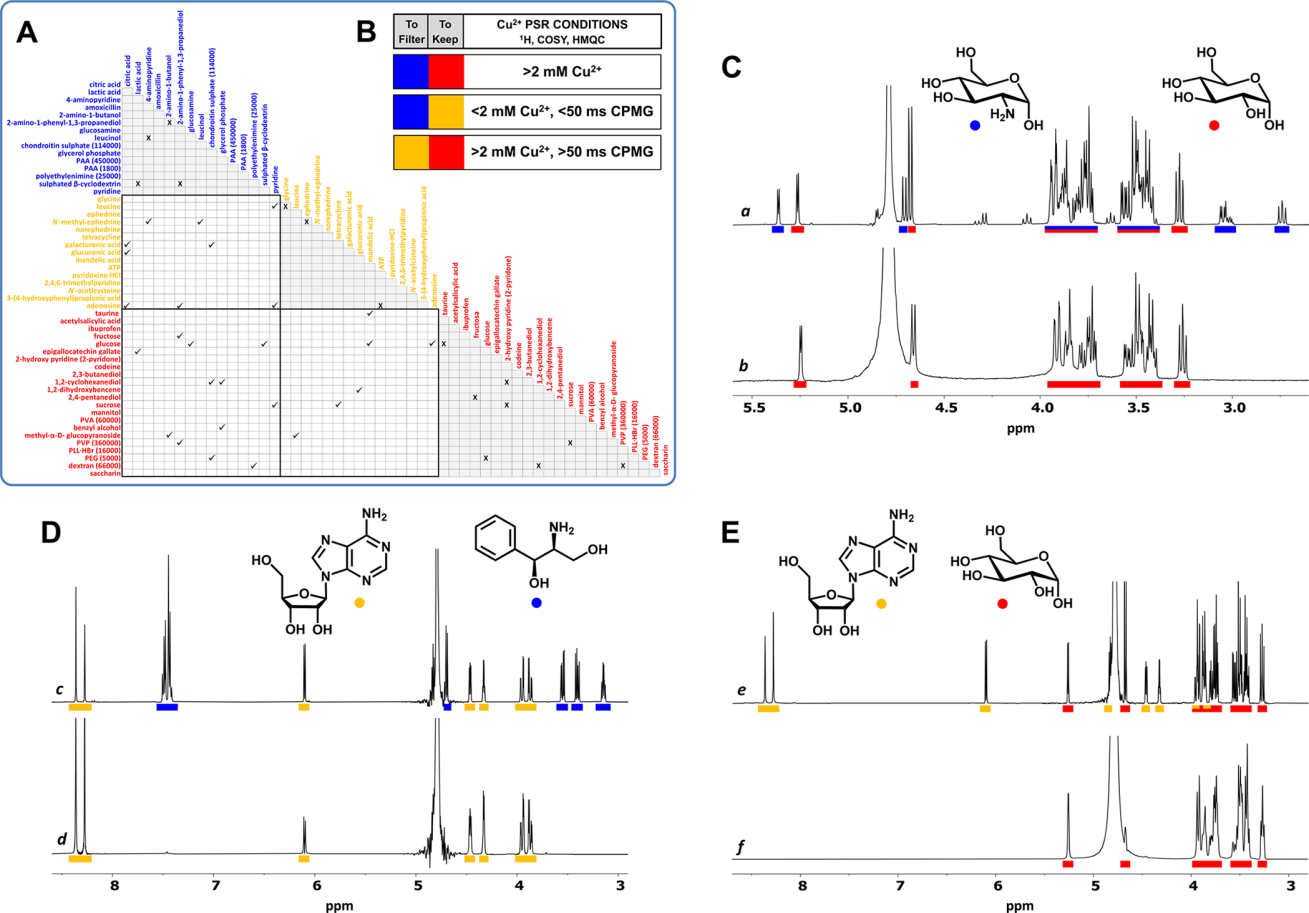
(A) Successful and failed PSR suppressions in two-component mixtures (D_2_O, 500 MHz). (B) PSR filtering conditions for selective ^1^H, COSY, and HMQC suppressions. Representative examples of selective suppressions between Blue-Yellow-Red categories. ^1^H NMR spectra (D_2_O, 500 MHz, 300 K) of a mixture of the following: (C) glucosamine (2 mg/mL) and glucose (2 mg/mL) before (a) and after (b) the addition of Cu^2+^ (2 mM), (D) 2-amino-1-phenyl-1,3-propanediol (1 mg/mL) and adenosine (3 mg/mL) before (c) and after (d) the addition of Cu^2+^ (0.16 mM) + *T*_2_-filter (CPMG, 30 ms), and (E) adenosine (1.2 mg/mL) and glucose (1.6 mg/mL) before (e) and after (f) the addition of Cu^2+^ (13 mM) + *T*_2_-filter (CPMG, 100 ms).

The scope of Cu^2+^ as coordinating PSR agent was assessed by analyzing the paramagnetic effect on the ^1^H NMR spectra of a collection of more than 50 small molecules and polymers of interest in the pharmaceutical and food industries, which display a large variety of functional groups. Depending on the extent of signal broadening (from no effect to complete suppression), these species were assigned to seven groups (Table 1), with the more sensitive ones comprising highly coordinating compounds. ^1^H NMR spectra of representative molecules in the upper, medium, and bottom parts of Table 1, recorded in the absence/presence of Cu^2+^, are shown in Figures S1–S3: (*1S*,*2S*)-2-amino-1-phenyl-1,3-propanediol, adenosine, sucrose. As a rule of thumb, the broadening effect of Cu^2+^ on the ^1^H NMR spectra of pyridines, diamines, polyamines, and amino alcohols was considerably higher than with Gd^3+^,^4^ in consistency with a higher coordination ability.

**Table 1:**
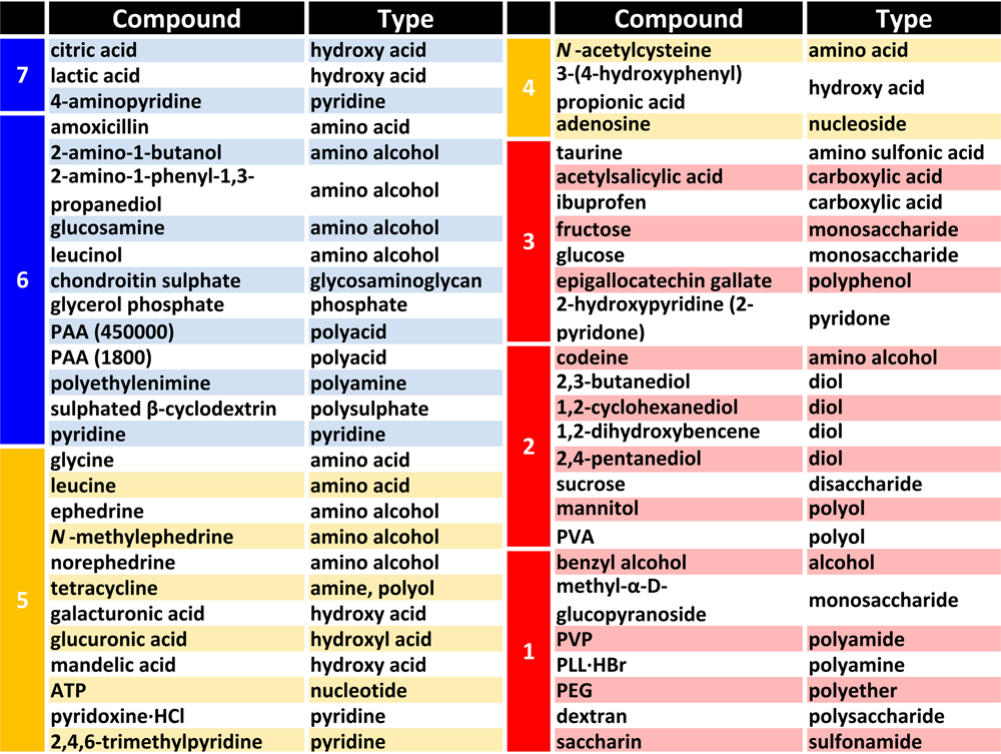
Paramagnetic Broadening Effect (Groups 1–7) and Ease of Suppression by Cu^2+^ (Blue, Yellow, Red Categories) in ^1^H NMR (4 mg/mL in D_2_O, 500 MHz, 300 K)^a^

a Group 7: suppression of all signals at <1 mM Cu^2+^, Group 6: suppression of all signals at >1 mM Cu^2+^, Group 5: broadening of all signals at >1 mM Cu^2+^, Group 4: suppression of some signals at >1 mM Cu^2+^, Group 3: broadening of some signals at >1 mM Cu^2+^, Group 2: reduced signal resolution at >1 mM Cu^2+^, Group 1: no effect on signal resolution at >1 mM Cu^2+^.

Next, the possibility of performing selective suppressions by Cu^2+^ among the seven groups in Table 1 was assessed in two-component mixtures (Figure 2A). It was confirmed that the more distant the groups, the easier the selective suppressions. Also, the impossibility of performing suppressions within a single group and between some neighboring groups. As a result, the initial groups (reflecting the paramagnetic broadening effect) were reduced to just three categories (designated as Blue, Yellow, and Red), according to their ease of suppression by Cu^2+^. In addition, from data in Figure 2A, general conditions for selective ^1^H, COSY, and HMQC suppressions between categories were determined (concentration of Cu^2+^/length of a complementary CMPG *T*_2_-filter; Figure 2B):

- Blue-Red: > 2.0 mM Cu^2+^
- Blue-Yellow: < 2.0 mM Cu^2+^, < 50 ms CPMG
- Yellow-Red: > 2.0 mM Cu^2+^, > 50 ms CPMG

Figure 2 depicts representative examples of successful suppressions within these three categories. For instance, the aforementioned glucosamine/glucose suppression can be easily rationalized now, considering their respective inclusion into the Blue and Red categories (Figure 2C). Similarly, Figure 2D,E show selective suppressions that exploit the use of complementary *T*_2_-filters between components of contiguous categories. Remarkably, none of these suppressions could be realized with Gd^3+^ as PSR ion (Figures S4–S6), confirming the coordination ability of Cu^2+^ as responsible of the selectivity achieved. Other representative spectra of successful (different categories) and unfruitful (same category) suppressions in the two-component mixtures shown in Figure 2A are included in the SI (Figures S7–S12). Additional advantages that emerged on the use of Cu^2+^ vs Gd^3+^ as PSR agent include the possibility of working in a wider pH-range (Gd^3+^ tends to precipitate at pH > 7.0) and a smaller broadening effect over the residual HOD signal.

The reliability of Table 1 to predict Cu^2+^ PSR filters in complex mixtures was challenged with a test bed of commercial drugs, namely (i) an amoxicillin/clavulanic acid antibiotic (amoxicillin, clavulanic acid, PEG, PVP); (ii) Cariban, a drug to treat nausea and vomiting in pregnancy (doxylamine, pyridoxine, sucrose); (iii) the antibiotic Proderma (doxycycline, sucrose); and (iv) Atepodin, a medicine for the treatment and diagnosis of supraventricular tachycardia (adenosine triphosphate, glycine, benzyl alcohol).

We started analyzing Amoxicillin/Clavulanic acid Cinfamed, an antibiotic that contains two active ingredients, the penicillin-like antibiotic amoxicillin (Blue) and the beta-lactamase inhibitor clavulanic acid (not included in Table 1 but expected to be Blue by similarity). NMR-visible excipients comprise poly(ethylene glycol) (PEG) and polyvinylpyrrolidone (PVP), both Red species. Attending to the Blue and Red categories of the constituents, a selective PSR suppression was anticipated for amoxicillin and clavulanic acid in the presence of Cu^2+^. Figure 3 shows their selective filtering, confirming the predictive character of Table 1, and the three color categories proposed. When lower concentrations of Cu^2+^ were assessed, it was even possible to attain a stepwise suppression of the Blue components, filtering first the more coordinating amoxicillin. Interestingly, the use of Gd^3+^ did not afford a clean suppression of any component, confirming Cu^2+^ as a PSR agent with characteristic filtering properties.

**Figure 3:**
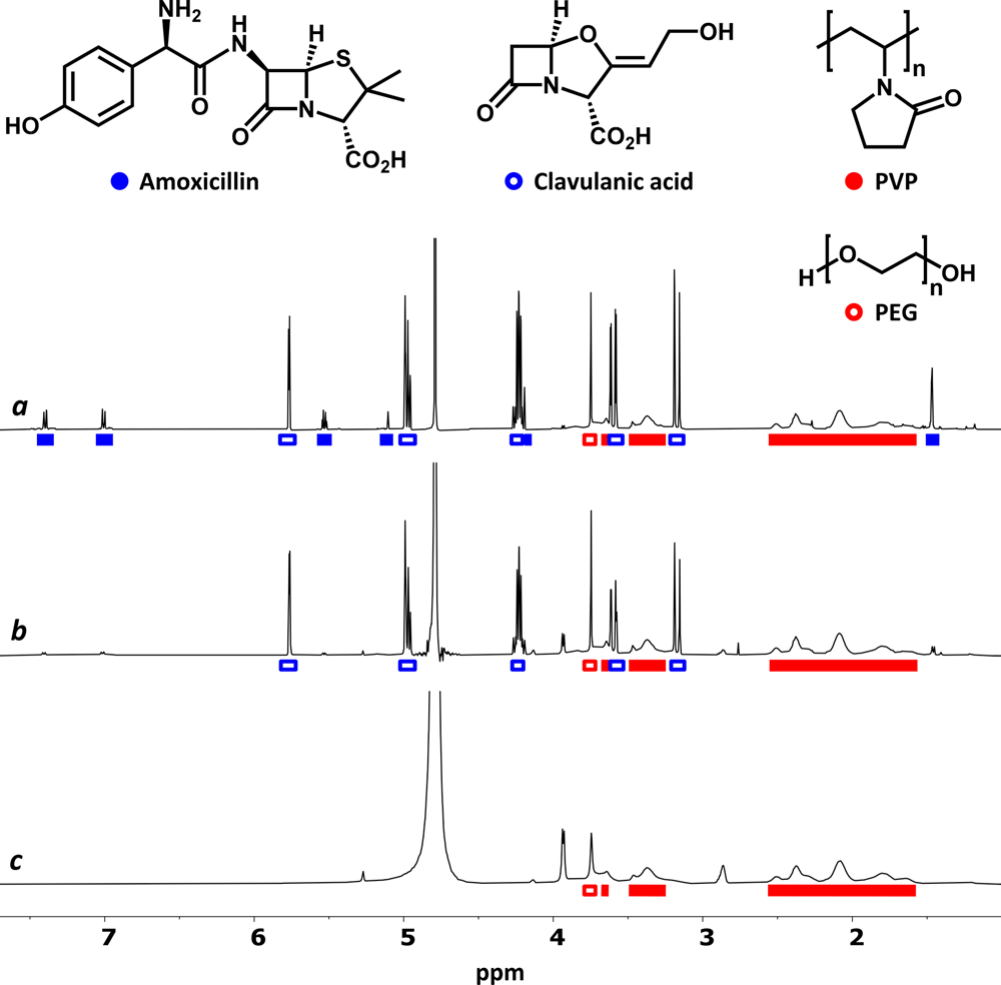
^1^H NMR spectra (D_2_O, 500 MHz, 300 K) of Amoxicillin/Clavulanic acid Cinfamed (10 mg/mL) before (a) and after the addition of 4 and 17 mM Cu^2+^ (b and c, respectively).

Then, we proceeded to analyze three commercial drugs (Cariban, Proderma, Atepodin) containing Yellow and Red components, where a complementary CPMG filter was expected for successful suppressions. Cariban is a medicine used to treat nausea and vomiting in pregnant women that contains doxylamine (antihistamine) and pyridoxine (vitamin B_6_) as active ingredients, both substituted pyridines that belong to the Yellow category. The mixture also includes sucrose (Red) as NMR-visible excipient. As predicted, the only addition of Cu^2+^ did not result in a clean filtering of doxylamine and pyridoxine. However, concomitant application of a short CPMG filter afforded their clean suppression, leaving unaffected the resolution and chemical shift of the sucrose signals (Figure 4). Remarkably, the fidelity of the PSR-CPMG filter was also demonstrated in 2D COSY and HMQC experiments, where the CPMG sequence was used as an excitation block replacing the first excitation pulse.^15^ Not unexpectedly, the use of Gd^3+^ as PSR agent was again unsuccessful, either in the absence or presence of CPMG filters.

**Figure 4:**
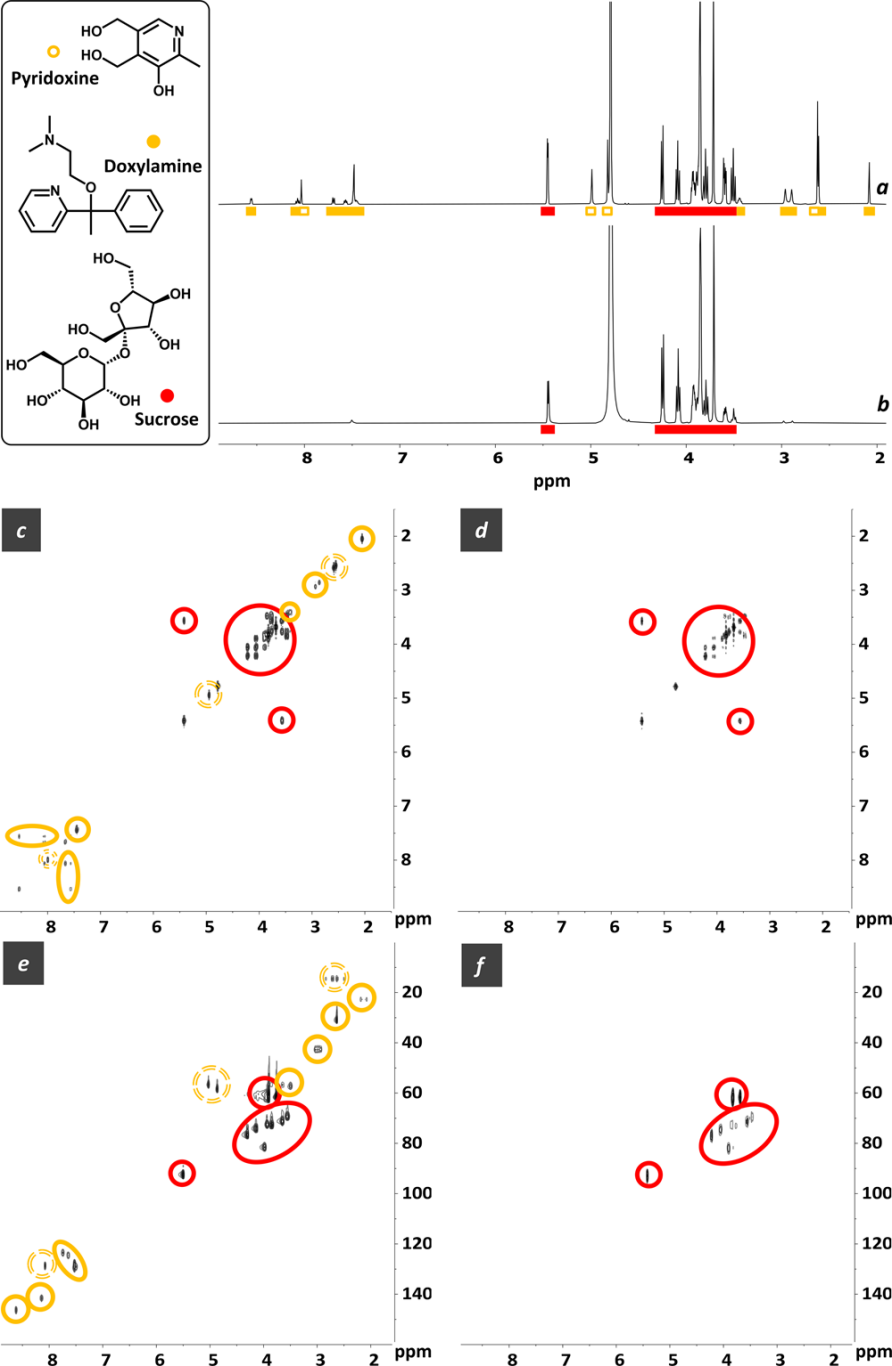
^1^H, ^1^H–^1^H COSY, and ^1^H–^13^C HMQC spectra (D_2_O, 500 MHz, 300 K) of Cariban (72 mg/mL) before (a, c, e) and after (b, d, f) the addition of Cu^2+^ (13 mM) + *T*_2_-filter (CPMG, 75 ms).

The antibiotic Proderma contains two NMR-visible components, the active ingredient doxycycline (Yellow) and sucrose (Red) as excipient. Here again, the direct filtering of the most sensitive Yellow component was unfeasible by the sole addition of Cu^2+^. However, implementation of a simultaneous short CPMG filter allowed the clean suppression of doxycycline (Figure 5). Very similar filtering conditions also allowed the efficient filtering of the Yellow components (adenosine triphosphate and glycine) of Atepodin (Figure S13).

**Figure 5:**
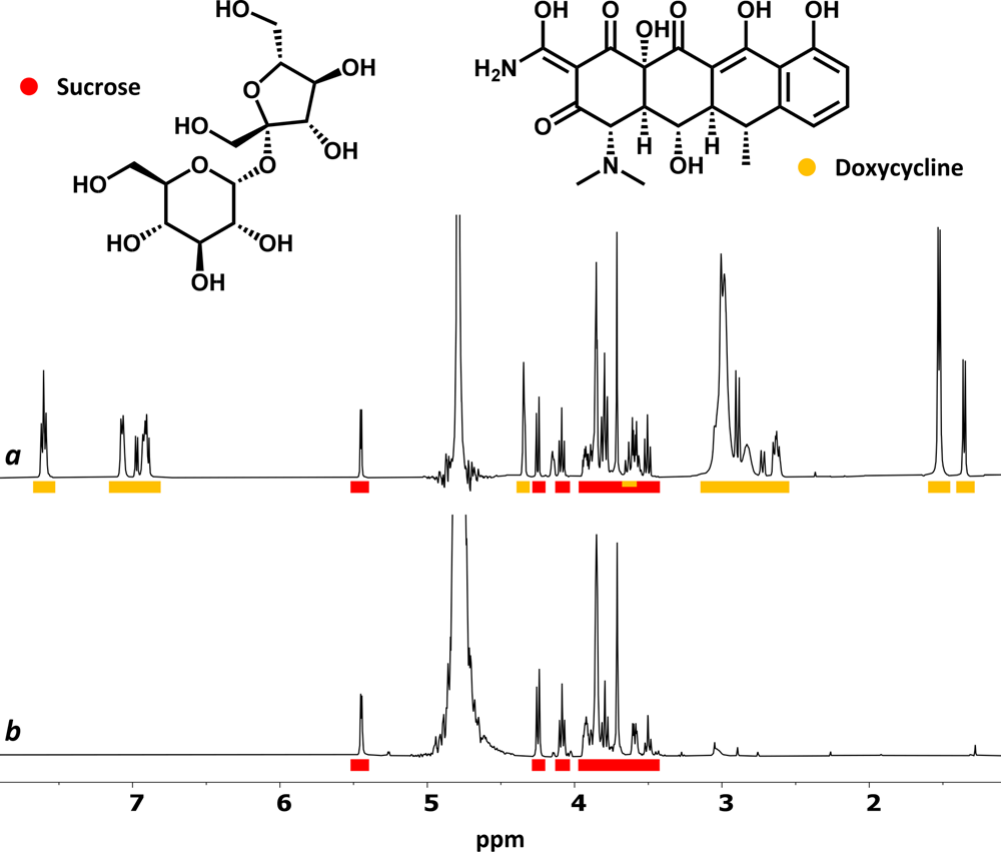
^1^H NMR spectra (D_2_O, 500 MHz, 300 K) of Proderma (4 mg/mL) before (a) and after (b) the addition of Cu^2+^ (4 mM) + *T*_2_-filter (CPMG, 75 ms).

Having established the utility of Cu^2+^ as PSR agent with filtering properties dependent on the coordination ability of the components in a mixture (rather than ion-pair interactions for Gd^3+^), we decided to assess the integration of both paramagnetic ions in the filtering of complex mixtures. To this end, we selected Acetilcisteina Mylan, a commercial mucolytic drug composed of acetylcysteine (^Cu^Yellow/^Gd^Yellow) as active ingredient, citric acid (^Cu^Blue/^Gd^Blue) as excipient, and two sweeteners, D-mannitol (^Cu^Red/^Gd^Yellow) and sodium saccharin (^Cu^Red/^Gd^Red). Attending to the Blue-Yellow-Red category of the components toward Gd^3+^, we have previously reported the sequential suppression of citric acid in a first step, followed by the simultaneous suppression of acetylcysteine and D-mannitol (both ^Gd^Yellow components).^4^ Herein, the stronger Cu^2+^-coordination of acetylcysteine than D-mannitol (Table 1) has been exploited for the stepwise suppression of the three components in a way unattainable with a single PSR agent. Thus, as shown in Figure 6, after an initial suppression of citric acid with Gd^3+^, acetylcysteine was filtered with Cu^2+^, followed by a final suppression of D-mannitol with Gd^3+^.

**Figure 6:**
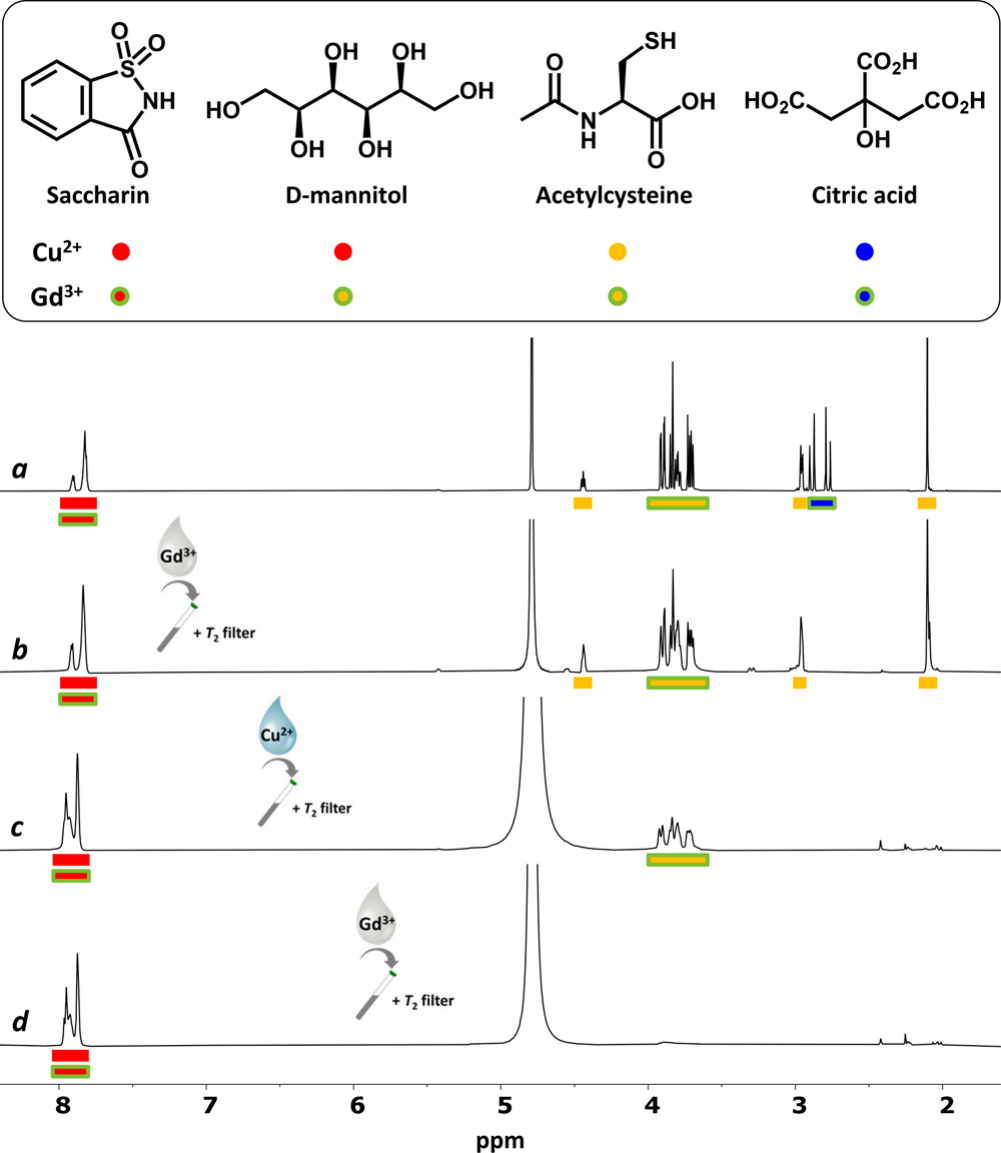
NMR-visible components of Acetilcisteina Mylan and their respective Blue-Yellow-Red categories toward Cu^2+^ and Gd^3+^. ^1^H NMR spectra (D_2_O, 500 MHz, 300 K) of Acetilcisteina Mylan (25 mg/mL) supplemented with saccharin (12.5 mg/mL) before (a) and after the addition of (b) Gd^3+^ (25 μM) + *T*_2_-filter (CPMG, 25 ms), (c) Cu^2+^ (2.5 mM) + *T*_2_-filter (CPMG, 250 ms), and (d) Gd^3+^ (0.2 mM) + *T*_2_-filter (CPMG, 300 ms).

Application of the PSR filter with Cu^2+^ starts with the assignment of the individual components in a mixture to the Blue-Yellow-Red categories. As a first approach, users are advised to find structural similarities between the species in a mixture of interest and those in Table 1. Nevertheless, for a proper inclusion of species in the Blue-Yellow-Red categories, the paramagnetic effect of Cu^2+^ on their ^1^H NMR spectra should be determined as described in Table 1 (extent of signal broadening as a function of the concentration of Cu^2+^). Once the species have been assigned to the three categories, selective suppressions could be expected by application of the PSR conditions shown in Figure 2B. While clean suppressions operate for Blue species in the presence of Red ones by the simple addition of mM concentrations of Cu^2+^, the selective filtering of species from contiguous categories (Blue-Yellow and Yellow-Red) are unfeasible by the sole addition of Cu^2+^, being necessary the implementation of simultaneous CPMG filters. Although the selective filtering of species within a category might work in specific examples using increasing concentrations of Cu^2+^ or tuning the length of CPMG filters, this will not be of general application because the ^1^H *T*_2_ values of the species in a category will level down in the presence of a PSR agent, making unlikely their selective suppression.

In conclusion, Cu^2+^ is presented as a robust PSR agent with characteristic NMR filtering properties different than Gd^3+^, the archetypal PSR agent so far. Not only do the paramagnetic properties change between nuclei, but also their complexation modes differ, offering the opportunity to tune the outcome of the PSR filter. While Gd^3+^ relies on the ion-pair complexation ability of the components in a mixture (mainly anionic species), Cu^2+^ stands out because of a greater capacity of filtering species that participate in coordination complexes, such as pyridines, diamines, polyamines, and amino alcohols. An evaluation of the paramagnetic effect of Cu^2+^ over more than 50 small molecules and polymers has unveiled three categories of compounds (Blue-Yellow-Red categories according to their ease of suppression by Cu^2+^) and precise filtering conditions for ^1^H, COSY, and HMQC between them. The integration of the specific filtering properties of Cu^2+^ and Gd^3+^ as PSR agents to the analysis of complex mixtures has been also demonstrated, widening the horizons of the PSR technology to quality control, natural product extracts, or the metabolic profiling of biological samples. Finally, having demonstrated the utility of Cu^2+^ as PSR agent, a more precise assignment of species to the Blue-Yellow-Red categories is envisaged using the transverse relaxation enhancement (*R*_2p_), as previously done for Gd^3+^.^4^ This approach, which involves the analysis of the^1^H *T*_2_ values of the species of interest in the absence and presence of Cu^2+^, will be the focus of our investigations in the future and reported in due time.

## Supporting Information Available

Materials, methods, and NMR spectra (PDF)
