# Supplementary material for: Filtering the NMR Spectra of Mixtures by Coordination to Paramagnetic Cu2+
Source: Anal Chem. 2022 Jul 27;94(31):10907–11. doi: 10.1021/acs.analchem.2c01983 (PMC9366733; doi:10.1021/acs.analchem.2c01983)
Supplement: Supplementary file 1 — ac2c01983_si_001.pdf [file ac2c01983_si_001.pdf]

Supporting Information for:

Filtering the NMR Spectra of Mixtures by Coordination to Paramagnetic Cu^{2+}

Juan Correa, Ana Garcia-Barandela, Llorenç Socias-Pinto, and Eduardo Fernandez-Megia*

ef.megia@usc.es

Centro Singular de Investigación en Química Biolóxica e Materiais Moleculares (CIQUS), Departamento de Química Orgánica, Universidade de Santiago de Compostela, Jenaro de la Fuente s/n, 15782 Santiago de Compostela, Spain.

Table of Contents

1. Materials S3

2. Sample Preparation S4

3. NMR Spectroscopy S4

4. NMR Spectra S6

5. References S19

1. Materials

All chemicals were purchased from commercial sources and used without further purification. $CuSO_4 \cdot 5H_2O$ was purchased from Prolabo. $Gd_2(SO_4)_3 \cdot 8H_2O$ was purchased from Aldrich. Dextran from *Leuconostoc mesenteroides* (M_n 33698, M_w 65794, by GPC), polyvinylpyrrolidone (PVP) (M_w 360000), citric acid, glucuronic acid, poly-L-lysine hydrobromide (PLL) (M_n 12400, M_w 16100, by MALLS), D-galacturonic acid monohydrate, 2,4,6-trimethylpyridine, and codeine were purchased from Fluka. Methyl α-D-glucopyranoside, sucrose, L-(–)-norephedrine, acetylsalicylic acid, taurine, saccharin, D-glucosamine hydrochloride, and chondroitin sulfate sodium salt (M_n 66784, M_w 114098, by GPC) were obtained from Sigma. L-leucine, (*R*)-(–)-leucinol, benzyl alcohol, (*R*)-(–)-2-amino-1-butanol, (1*S*,2*S*)-(+)-2-amino-1-phenyl-1,3-propanediol, 2-hydroxy-pyridine, 3-(4-hydroxyphenyl)-propionic acid, (1*S*,2*S*)-*trans*-1,2-cyclohexane diol, *meso*-2,3-butanediol, (1*S*,2*R*)-(+)-*N*-methylephedrine, polyethylenimine branched (M_n 10000 by GPC, M_w 25000 by LS), and polyacrylic acid (PAA) [PAA_{450000} (M_v 450000) and PAA_{1800} (M_n 1022, M_w 1773)] were purchased from Aldrich. D-Mannitol, ibuprofen, 4-aminopyridine, tetracycline, D-(+) glucose, lactic acid, (–)-epigallocatechin gallate, 1,2 dihydroxybenzene, acetylcysteine, glycine, and sulfated β-cyclodextrin were purchased from Sigma-Aldrich. 2,4-Pentanediol, adenosine, and adenosine 5'-triphosphate sodium salt were purchased from Merck. Polyvinyl alcohol (PVA) (M_w 60000 by GPC), and β-glycerol phosphoric acid disodium salt were obtained from Acros. Pyridine was purchased from Fisher Scientific. Fructose was obtained from Azaconsa. (+)-Ephedrine hydrochloride was purchased from Ega-Chemie. Pyridoxine hydrochloride was obtained from BLDpharm. Amoxicillin/Clavulanic acid 500/125 mg was obtained from Cinfa. Proderma 60 mg was obtained from IFC (Industrial Farmacéutica Cantabria, S. A.). Cariban 10 mg/10

mg was obtained from Inibsa Ginecología. Acetylcysteine Mylan 600 mg was obtained from Mylan Pharmaceuticals.

2. Sample Preparation

Two-component mixtures for NMR analysis were prepared at concentrations in the range 0.1-4.0 mg/mL. Relative molar ratios of the components were selected to ensure comparable intensity of their signals in the NMR spectra. Commercial samples were prepared as shown below:

- Amoxicillin/Clavulanic acid: a tablet of was grinded and suspended in D_2O (5.0 mL). After stirring for 2 h, the suspension was filtered through a nylon filter (0.45 mm) and the filtrate was lyophilized. The residue was dissolved in D_2O at the indicated concentration.
- Proderma: a tablet was grinded and suspended in D_2O (2.0 mL). After stirring for 1 h, the suspension was filtered through a nylon filter (0.45 mm) and the filtrate was lyophilized. The residue was dissolved in D_2O at the indicated concentration.
- Cariban: a tablet was grinded and suspended in D_2O (2.0 mL). After stirring for 10 min, the suspension was filtered through a nylon filter (0.45 mm) and the filtrate was lyophilized. The residue was dissolved in D_2O at the indicated concentration.
- Mylan was dissolved directly in D_2O at the indicated concentration.

3. NMR Spectroscopy

^{1}H NMR spectra of mixtures were recorded on a Bruker DRX 500 MHz spectrometer. 2D NMR spectra were recorder on a Bruker Avance DRX 500 MHz spectrometer with field strength 11.7 T, equipped with an inverse detection ^{1}H/X broadband BBI probe with z gradients and operated with top-spin 1.3 software. Chemical shifts (δ) are

reported in ppm relative to the residual water peak (HOD, δ=4.79 ppm) used as an internal standard. ^1H-^1H COSY spectra were recorder in magnitude mode using the standard Bruker sequence "cosygp". ^1H-^{13}C HMQC experiments were recorded using the standard Bruker sequence "inv4gp". 1D and 2D T_2-edited (Carr-Purcell-Meiboom-Gill, CPMG) experiments were performed by replacing the first 90° pulse by the CPMG pulse sequence as previously described,[1,2] using a τ value of 0.7 ms. MestReNova 14.2 software (Mestrelab Research) was used for spectral processing. When comparing spectra, the same number of scans and apodization values were used. $CuSO_4 \cdot 5H_2O$ was used as source of Cu^{2+}.

4. NMR Spectra

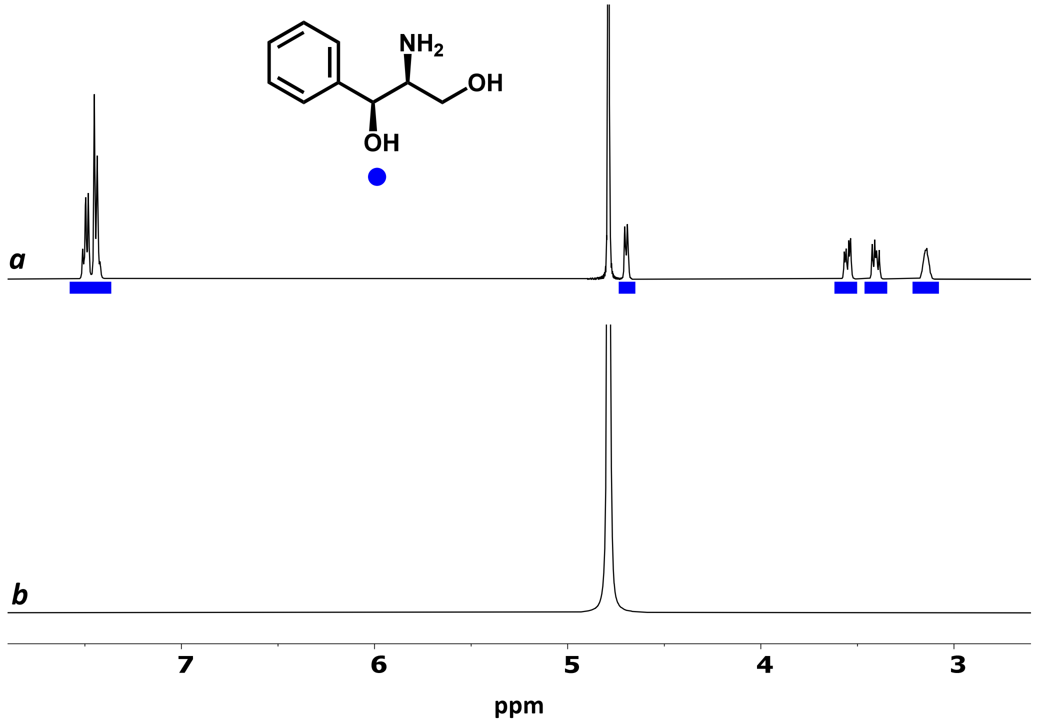

Figure S1. ^{1}H NMR spectra (D_2O, 500 MHz, 300 K) of (1*S*,2*S*)-2-amino-1-phenyl-1,3-propanediol (4 mg/mL) before (a) and after (b) the addition of Cu^{2+} (2 mM).

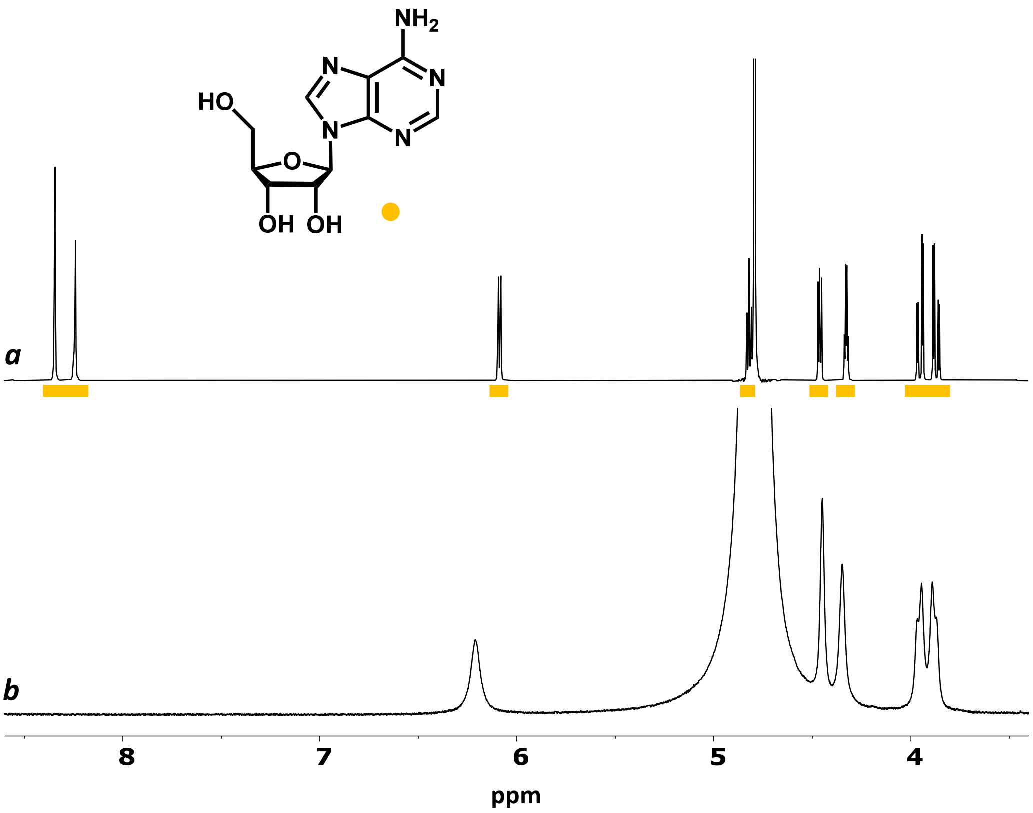

Figure S2. ^{1}H NMR spectra (D_2O, 500 MHz, 300 K) of adenosine (4 mg/mL) before (a) and after (b) the addition of Cu^{2+} (20 mM).

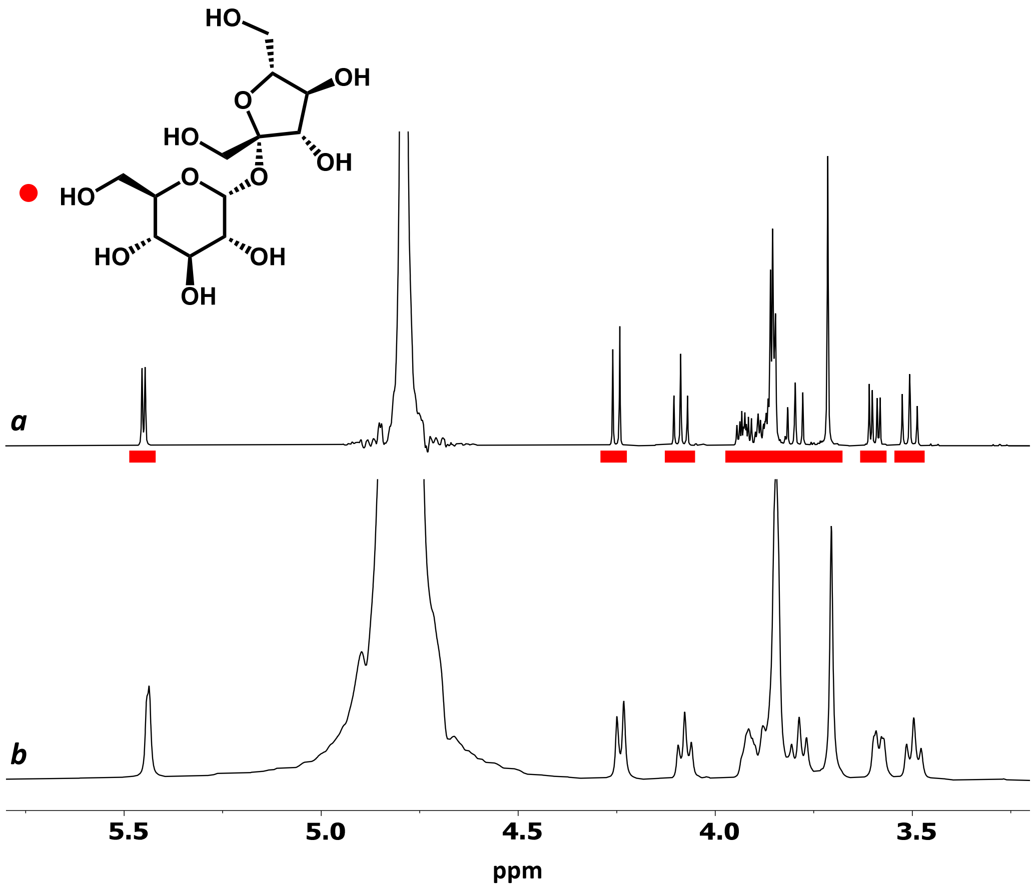

Figure S3. ^{1}H NMR spectra (D_2O, 500 MHz, 300 K) of sucrose (4 mg/mL) before (a) and after (b) the addition of Cu^{2+} (10 mM).

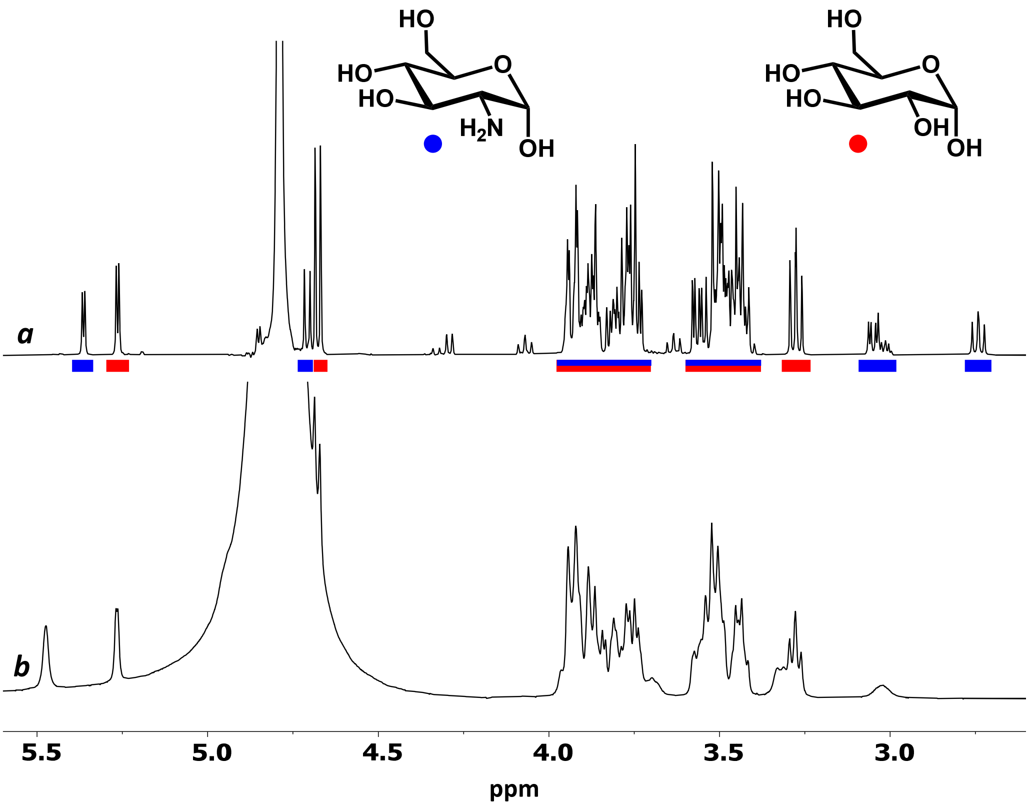

Figure S4. ^{1}H NMR spectra (D_2O, 500 MHz, 300 K) of a mixture of glucosamine (2 mg/mL) and glucose (2 mg/mL) before (a) and after (b) the addition of Gd^{3+} (0.6 mM). Note that the colour codes of the components refer to their ease of suppression by Cu^{2+}.[3]

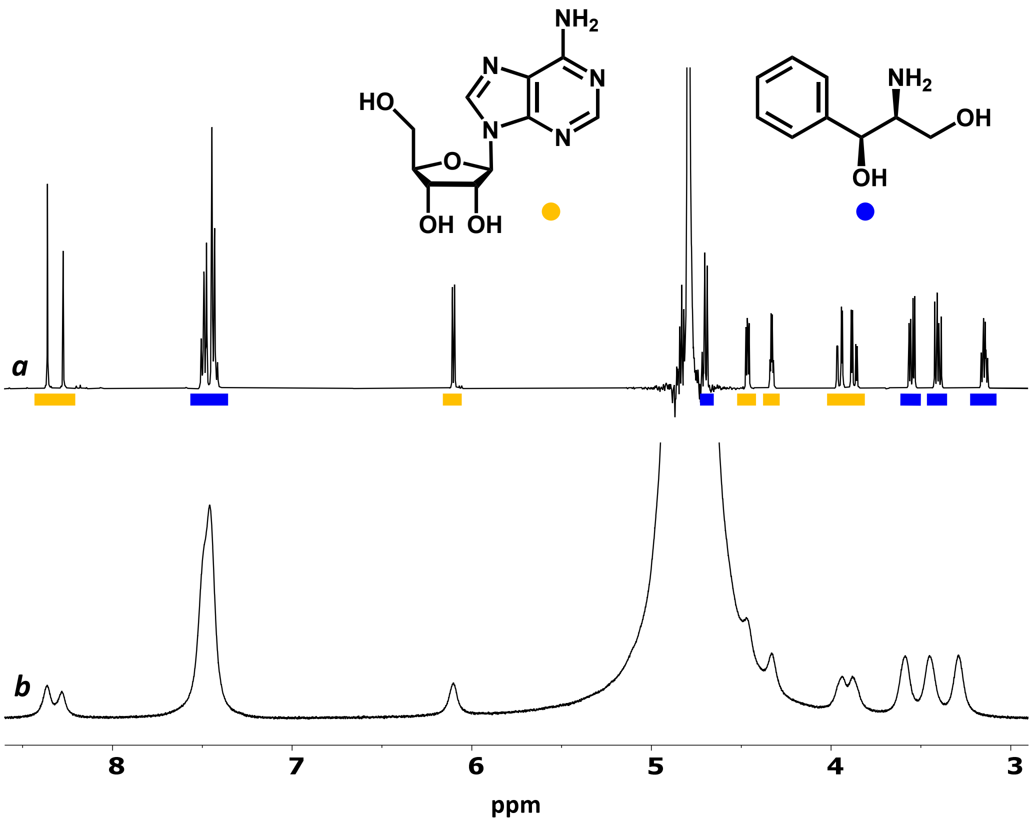

Figure S5. ^{1}H NMR spectra (D_2O, 500 MHz, 300 K) of a mixture of 2-amino-1-phenyl-1,3-propanediol (1 mg/mL) and adenosine (3 mg/mL) before (a) and after (b) the addition of Gd^{3+} (0.2 mM). Note that the colour codes of the components refer to their ease of suppression by Cu^{2+}.[3]

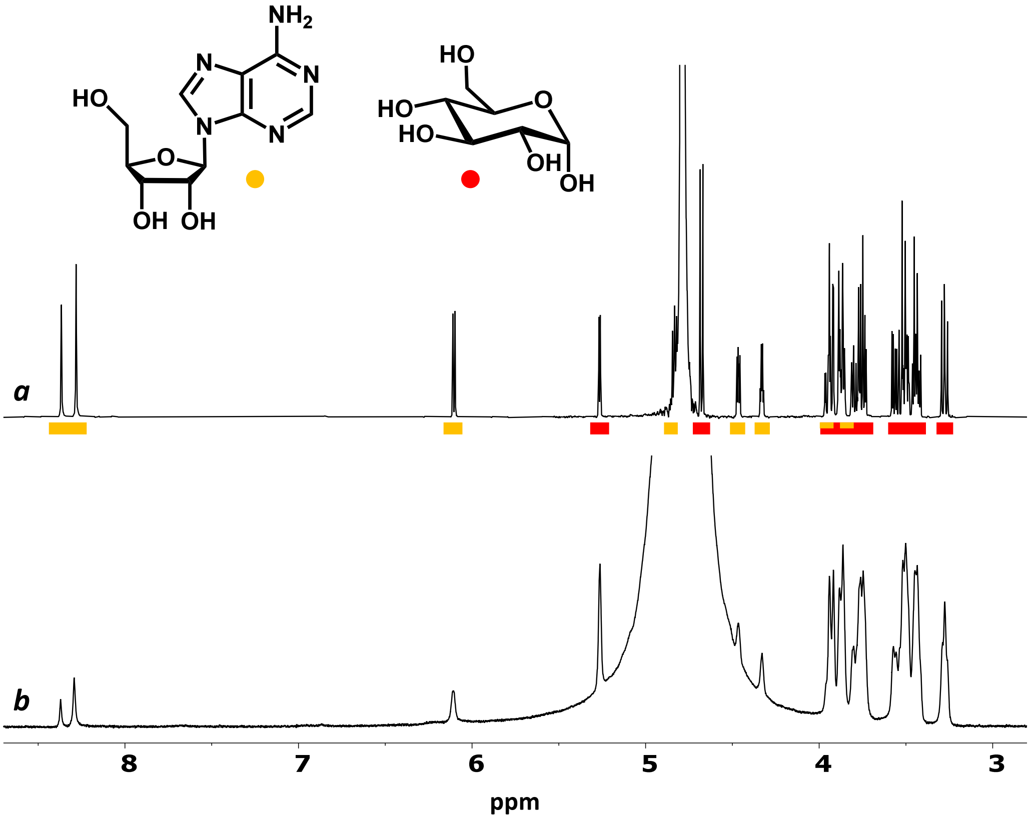

Figure S6. ^{1}H NMR spectra (D_2O, 500 MHz, 300 K) of a mixture of adenosine (1.2 mg/mL) and glucose (1.6 mg/mL) before (a) and after (b) the addition of Gd^{3+} (0.2 mM). Note that the colour codes of the components refer to their ease of suppression by Cu^{2+}.[3]

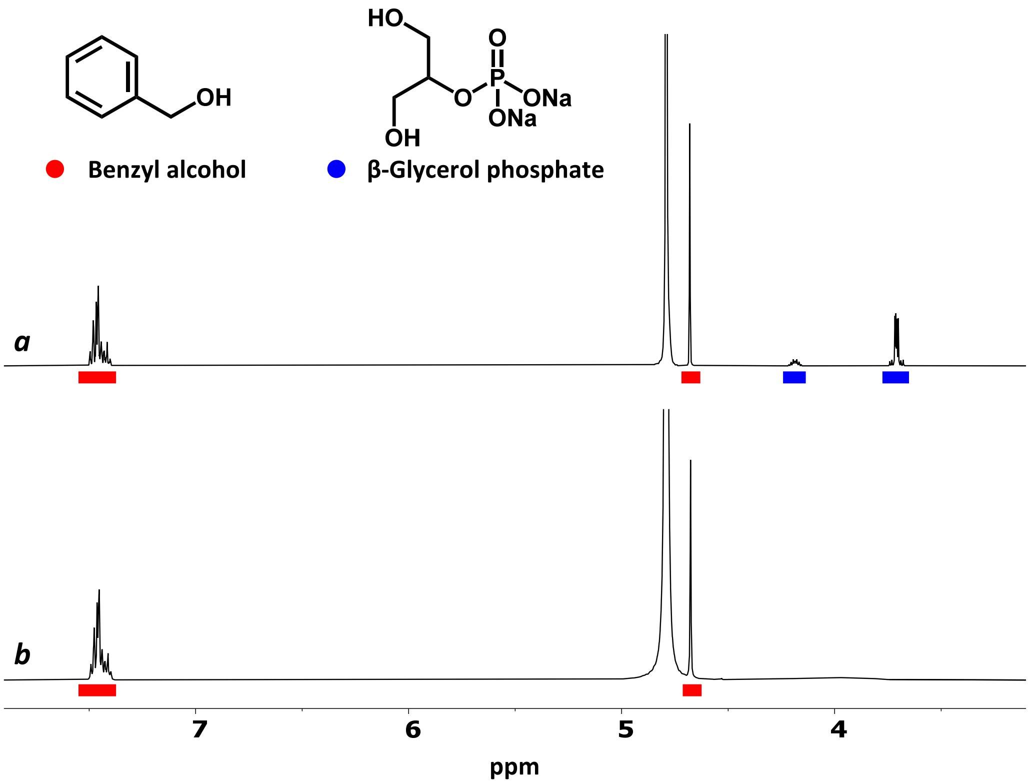

Figure S7. ^{1}H NMR spectra (D_2O, 500 MHz, 300 K) of a mixture of β-glycerol phosphate (1.6 mg/mL) and benzyl alcohol (2 mg/mL) before (a) and after (b) the addition of Cu^{2+} (2 mM).

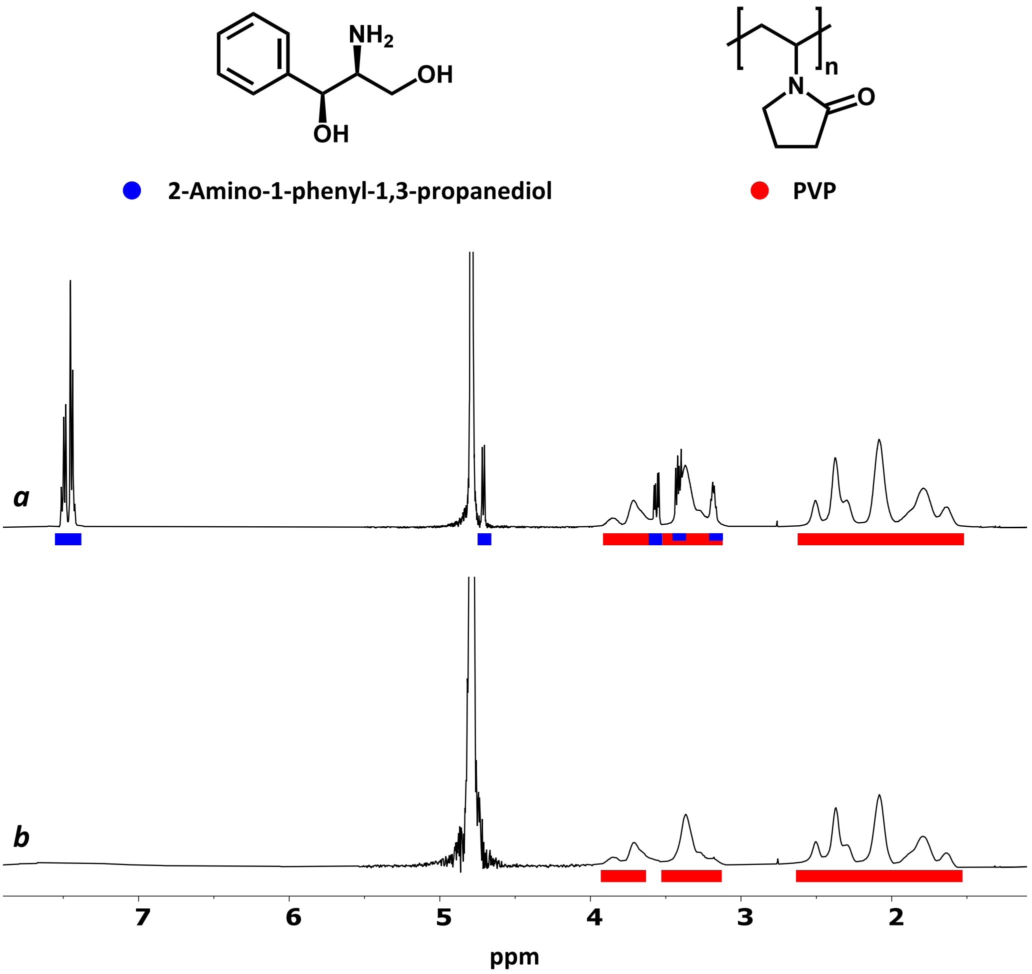

Figure S8. ^{1}H NMR spectra (D_2O, 500 MHz, 300 K) of a mixture of 2-amino-1-phenyl-1,3-propanediol (1 mg/mL) and PVP (2 mg/mL) before (a) and after (b) the addition of Cu^{2+} (2 mM).

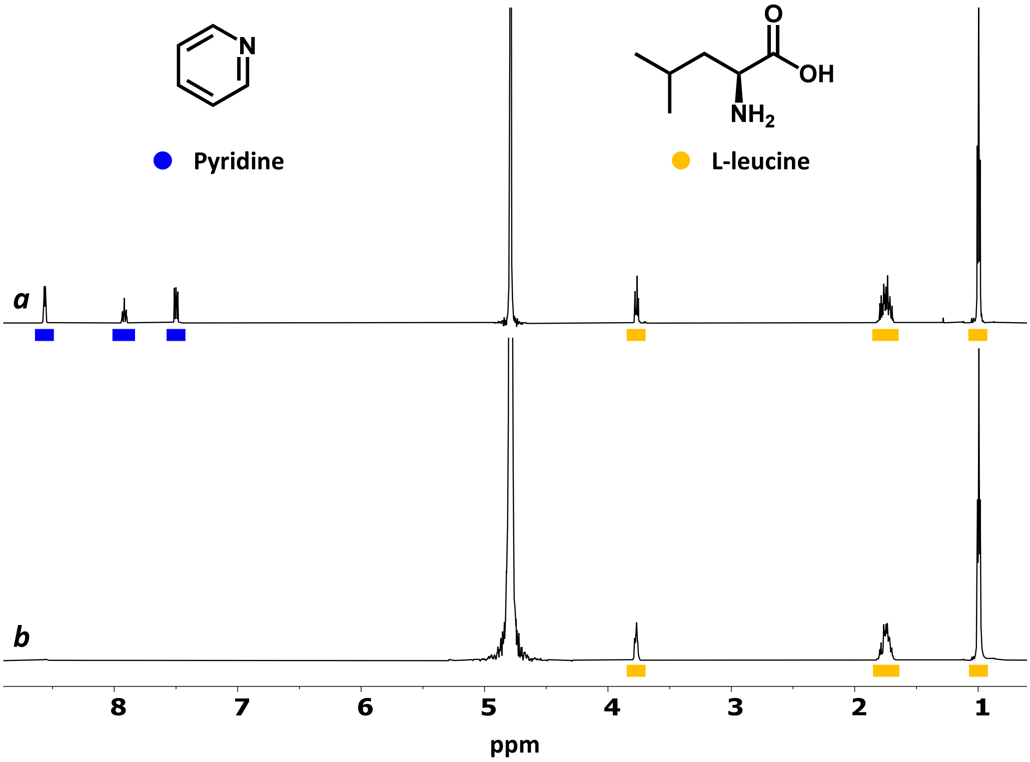

Figure S9. ^{1}H NMR spectra (D_2O, 500 MHz, 300 K) of a mixture of pyridine (0.8 mg/mL) and L-leucine (1.4 mg/mL) before (a) and after (b) the addition of Cu^{2+} (0.6 mM) + T_2-filter (CPMG, 30 ms).

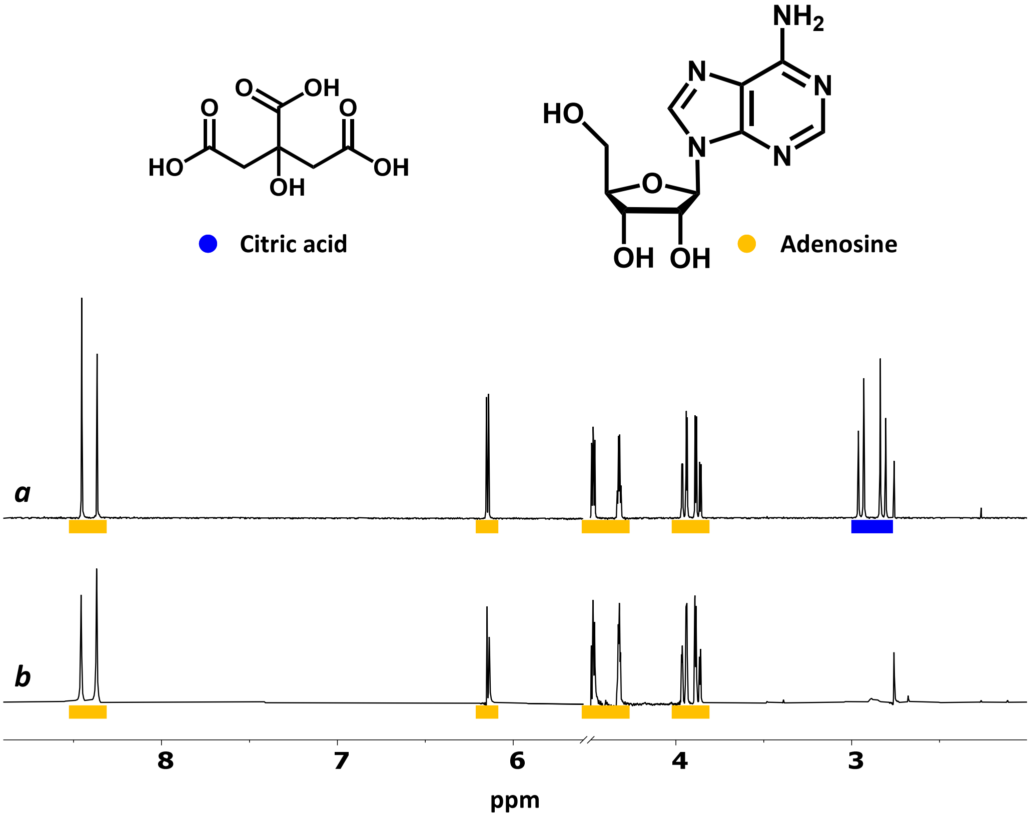

Figure S10. ^{1}H NMR spectra (D_2O, 500 MHz, 300 K) of a mixture of citric acid (0.1 mg/mL) and adenosine (2.4 mg/mL) before (a) and after (b) the addition of Cu^{2+} (0.05 mM) + T_2-filter (CPMG, 50 ms).

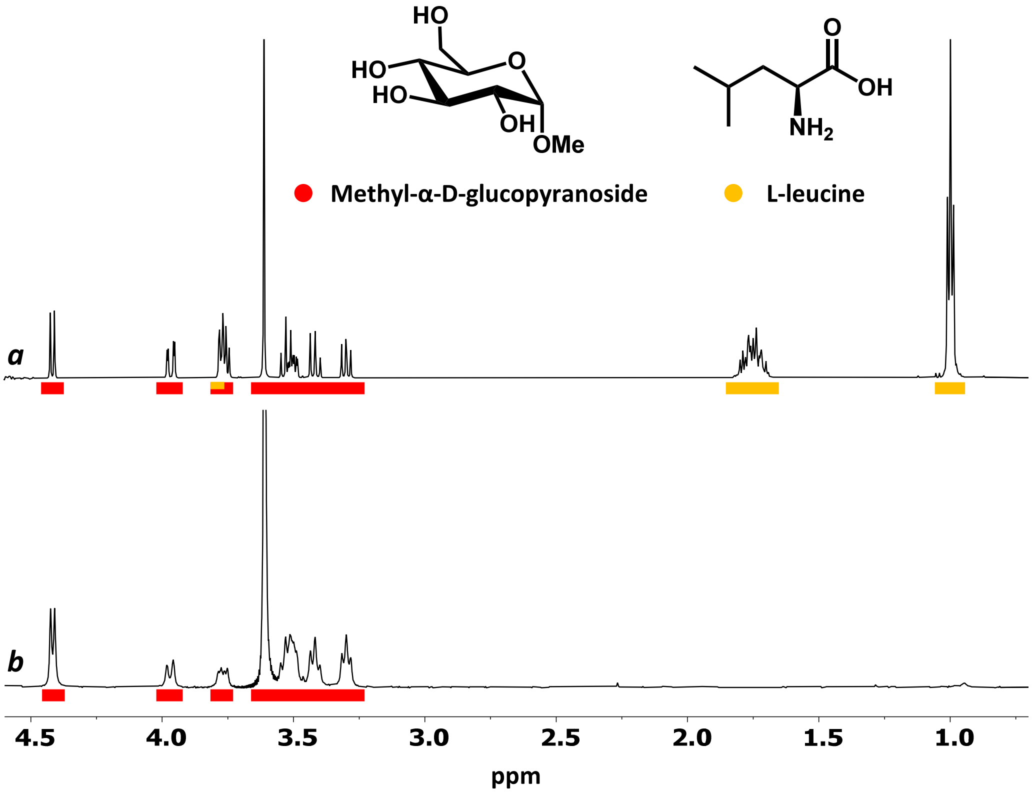

Figure S11. ^{1}H NMR spectra (D_2O, 500 MHz, 300 K) of a mixture of methyl-α-D-glucopyranoside (0.8 mg/mL) and L-leucine (1.4 mg/mL) before (a) and after (b) the addition of Cu^{2+} (36 mM) + T_2-filter (CPMG, 300 ms).

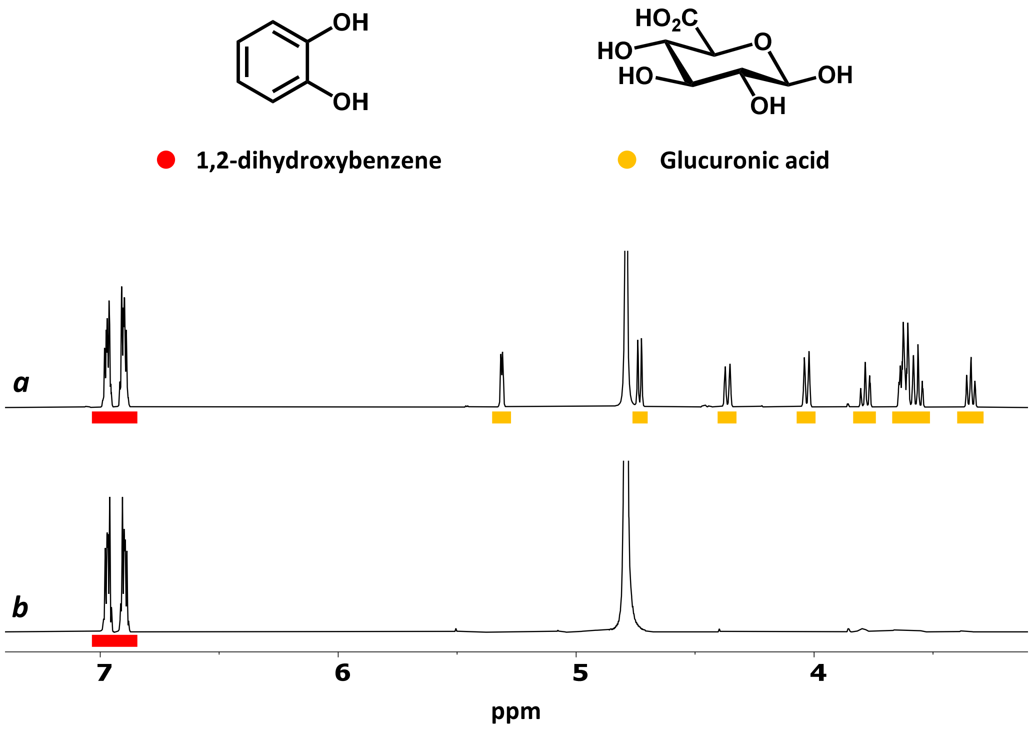

Figure S12. ^{1}H NMR spectra (D_2O, 500 MHz, 300 K) of a mixture of 1,2-dihydroxybenzene (2 mg/mL) and glucuronic acid (4 mg/mL) before (a) and after (b) the addition of Cu^{2+} (3.2 mM) + T_2-filter (CPMG, 100 ms).

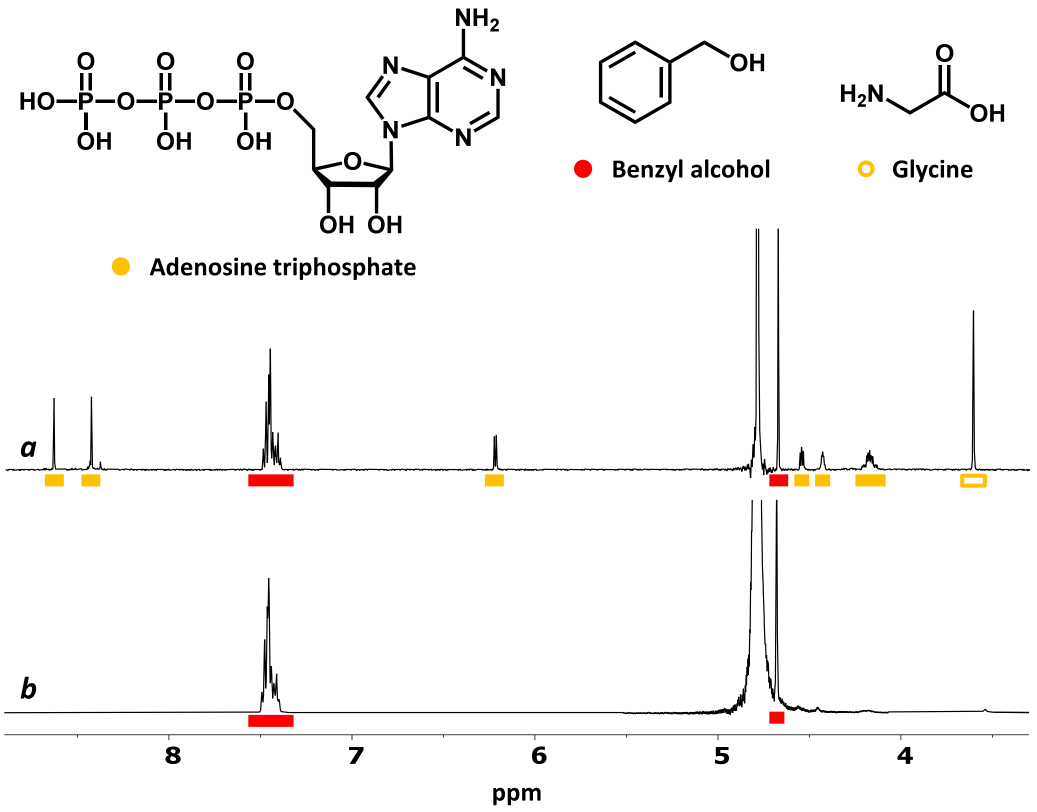

Figure S13. ^{1}H NMR spectra (D_2O, 500 MHz, 300 K) of a mixture of adenosine triphosphate (3.4 mg/mL), glycine (0.4 mg/mL), and benzyl alcohol (0.8 mg/mL) before (a) and after (b) the addition of Cu^{2+} (6 mM) + T_2-filter (CPMG, 100 ms).

5. References

1 . Meiboom, S.; Gill, D. Modified Spin-Echo Method for Measuring Nuclear Relaxation Times. *Rev. Sci. Instrum.* **1958**, *29*, 688-691.

2. Williams, P. G.; Saunders, J. K.; Dyne, M.; Mountford, C. E.; Holmes, K. T. Application of a T_2-filtered COSY Experiment to Identify the Origin of Slowly Relaxing Species in Normal and Malignant Tissue. *Magn. Reson. Med.* **1988**, *7*, 463-471.

3. Correa, J.; Pinto, L. F.; Riguera, R.; Fernandez-Megia, E. Predicting PSR Filters by Transverse Relaxation Enhancements. *Anal. Chem.* **2015**, *87*, 760-767.
